# Recent Trends in Medicaid Spending and Use of Drugs With US Food and Drug Administration Accelerated Approval

**DOI:** 10.1001/jamahealthforum.2021.3177

**Published:** 2021-10-08

**Authors:** Rachel E. Sachs, Kyle A. Gavulic, Julie M. Donohue, Stacie B. Dusetzina

**Affiliations:** 1 Washington University in St Louis School of Law, St Louis, Missouri; 2 Yale School of Medicine, New Haven, Connecticut; 3 Editorial Intern, *JAMA Health Forum*; 4 Department of Health Policy and Management, University of Pittsburgh Graduate School of Public Health, Pittsburgh, Pennsylvania; 5 Associate Editor, *JAMA Health Forum*; 6 Vanderbilt-Ingram Cancer Center, Nashville, Tennessee; 7 Department of Health Policy, Vanderbilt University School of Medicine, Nashville, Tennessee

## Abstract

**Importance:**

State Medicaid programs have reported concerns about rising drug prices and spending, particularly regarding drugs entering the market through the accelerated approval program under the US Food and Drug Administration (FDA). The accelerated approval program enables the FDA to approve drugs on the basis of unverified surrogate end points, meaning that clinical benefits for these products are uncertain at the time of approval. However, state Medicaid programs are legally required to cover these drugs. Little is known about the set of products with accelerated approval over time, their use among Medicaid beneficiaries, or the magnitude of their financial influence on state Medicaid programs.

**Objective:**

To identify the number and class of drugs approved through the FDA’s accelerated approval pathway and analyze state Medicaid programs’ use and spending on these drugs from 2015 through 2019.

**Design, Setting, and Participants:**

In this cross-sectional study, biannual FDA reports were used to identify products granted accelerated approval and their associated indications approved between December 1992 and December 2020. State Medicaid Drug Utilization Data files available for 1992 through 2019 were used to estimate national totals for spending and use of outpatient drugs.

**Main Outcomes and Measures:**

National Medicaid use and gross and net spending on drugs with accelerated approval from 2015 through 2019.

**Results:**

Since the inception of the FDA’s accelerated approval pathway in 1992 through 2020, 216 product-indication pairs granted accelerated approval were identified, comprising 149 unique products. The composition of drugs approved through the pathway has changed over time, with 28 of 30 (93.3%) product-indication pairs receiving accelerated approval in 2020 being indicated for cancer. Relative to all outpatient prescription drugs paid for by Medicaid, products with accelerated approval ranged from 0.2% to 0.4% of use (1.3-2.4 million prescriptions annually). Despite their infrequent use, drugs with accelerated approval represented a minimum annual net spending on all drugs covered by Medicaid of 6.4% ($2.2 billion of $34.6 billion) in 2015 and a maximum of 9.1% ($2.5 billion of $27.6 billion) in 2018. Estimated annual gross spending on drugs with accelerated approval ranged from $4.2 billion to $4.9 billion over 2015 through 2019, and estimated net spending from $2.2 billion to $2.6 billion.

**Conclusions and Relevance:**

In this cross-sectional study of 216 drugs granted accelerated approval, state spending on drugs approved through the FDA’s growing accelerated approval program represented an outsized amount of spending relative to use. Because drugs with accelerated approval have come to market on the basis of trials using surrogate end points, considerable amounts of this spending may have been attributable to products with unproven clinical benefits.

## Key Points

**Question:**

How much do prescription drugs approved through the US Food and Drug Administration’s accelerated approval program contribute to state Medicaid program spending?

**Findings:**

In this cross-sectional study of 216 drugs granted accelerated approval from 1992 through 2020, relative to all drugs paid for by Medicaid, products with accelerated approval comprised less than 1% of use. Despite their infrequent use, annual net spending on drugs with accelerated approval represented 6.4% to 9.1% of net spending on all drugs covered by Medicaid (in 2015 and 2018, respectively).

**Meaning:**

Medicaid spending on drugs with accelerated approval represents an outsized amount of spending relative to their use.

## Teaser

This cross-sectional study identifies the number and class of drugs approved through the US Food and Drug Administration’s accelerated approval pathway and analyzes state Medicaid programs’ use and spending on these drugs from 2015 through 2019.

## Introduction

State Medicaid programs have expressed increasing concern about rising prescription drug spending. Some states^1^ have expressed concern about constraints on the strategies available to them for cost containment under the Centers for Medicare & Medicaid Services (CMS) Medicaid Drug Rebate Program, a system that has been in place since 1990. Under the rebate program, state Medicaid programs that choose to cover prescription drugs must cover essentially all drugs approved by the US Food and Drug Administration (FDA). In exchange, pharmaceutical companies must provide Medicaid with substantial discounts for drugs sold under the program.^2^ Because Medicaid cannot use traditional cost-control strategies such as closed or tiered formularies (unlike other insurers), this bargain was meant to protect states’ finances as they provide access to medically necessary drugs for their populations with Medicaid coverage.

A key area of concern for state Medicaid programs is the growing number of high-priced drugs entering the market through the FDA’s accelerated approval program (Table 1^3,4^).^5^ In 1992, the FDA established the accelerated approval program in response to the HIV/AIDS crisis, expediting the availability of drugs aiming to provide “meaningful therapeutic benefit compared to existing treatment” for illnesses considered “serious or life-threatening.”^6^^(p58942)^ For such drugs, the FDA grants early approval based on a surrogate end point rather than a clinical end point,^4^ on the condition that the manufacturer conducts postmarket studies to confirm a clinical benefit. Under the rebate program, state Medicaid programs remain legally obligated to cover these prescription drugs,^7^ although their approval is supported by less evidence than is typical for other drugs. Some of these drugs will turn out to have strong clinical benefits, as has been true with drugs used to treat HIV/AIDS approved under the program. But others will turn out to have no real clinical benefit for patients after decades of market access.^8,9^ Others may have list prices in the hundreds of thousands of dollars per year, with benefits uncertain for years to come.^10^ As a result, states and expert bodies have recently asked CMS to modify the link between FDA approval and mandatory coverage under the rebate program.^11,12^ Despite heightened interest in reforming the accelerated approval program or modifying payment related to these products, to our knowledge, no current studies have quantified Medicaid program spending on these drugs. In this cross-sectional study, we report the results of an analysis of state Medicaid spending on drugs with accelerated approval.

**Table 1.** Policies Promoting Innovation and Access to New Prescription Drugs

Policy	Supervising agency	Goal	Scope
Medicaid Drug Rebate Program	CMS	Increase patient access to prescription drugs while ensuring preferred pricing benefits	Applies to FDA-approved outpatient drugs, with few enumerated exclusions such as drugs “used for cosmetic purposes”^3^
Accelerated approval program	FDA	Enable drugs meeting unmet medical needs to reach the market more quickly	Applies to products intended to treat a “serious or life-threatening disease,” where the product has an “effect on a surrogate endpoint that is reasonably likely to predict clinical benefit”^4^

Abbreviations: CMS, Centers for Medicare & Medicaid Services; FDA, US Food and Drug Administration.

## Methods

### Data Source and Sample

We used the biannual report released by the FDA’s Center for Drug Evaluation and Research (CDER) enumerating drugs receiving accelerated approval based on a surrogate end point to identify products that received accelerated approval between December 1992 and December 2020. This data source includes information on a product’s proprietary name, active ingredient, FDA receipt date, FDA approval date, indication, whether the application was for a novel or supplemental indication, its conversion-withdrawal status, and its full approval conversion-withdrawal date, where applicable. We included product-indication pairs approved through the accelerated approval pathway, excluding duplicative indications, new formulations of previously approved products (eg, tablets vs oral solution), and indications that were merely an extension to include pediatric populations. Each product-indication pair was reviewed and categorized by disease target, including cancer, HIV, and other conditions.

We used the State Medicaid Drug Utilization Data files available from CMS for 1992 through 2019 (the last complete available year at the time of analysis) to estimate national totals for spending and use of drugs dispensed in outpatient settings. For accelerated approval–related spending and use, we limited the list of drugs approved by CDER to those reimbursed through the Medicaid outpatient pharmacy benefit and excluded 5 products that had an indication that did not receive accelerated approval and that would dominate the market (eg, ciprofloxacin, a commonly used antibiotic that later received accelerated approval as a treatment for inhalational anthrax). We also excluded products for which a National Drug Code (NDC) was unavailable owing to recent FDA approval (n = 11). See eMethods in the Supplement for further details on completion of a crosswalk between these data sets, and the eFigure and eTable 1 in the Supplement for details regarding product exclusion.

Because this cross-sectional study used publicly available data released at the aggregate (rather than individual) level, it was exempt from institutional review board review. This study conforms to the Strengthening the Reporting of Observational Studies in Epidemiology (STROBE) reporting guideline.

### Outcome Measures

The primary outcomes of interest were national Medicaid use and gross and net (postrebate) spending on drugs that received accelerated approval from 2015 through 2019. We focused on 2015 through 2019 for analysis owing to fundamental changes to Medicaid under the Affordable Care Act, including Medicaid expansion prior to 2015. We determined annual gross and net Medicaid spending on drugs with accelerated approval, the number of filled prescriptions of drugs with accelerated approval, and the annual percentage of total Medicaid outpatient drug use and spending dedicated to these products. We summarized gross Medicaid spending for drugs with accelerated approval by year based on reported national totals of total amount reimbursed in the Medicaid Drug Utilization Data files. To estimate net spending for drugs with accelerated approval, we first applied a minimum rebate of 23.1% (as required by statute) to all drugs with accelerated approval, except those indicated for blood clotting or approved exclusively for pediatric indications, for which we applied a 17.1% minimum statutory rebate. Next, we identified the first year each drug was observed and estimated the median unit price at the 11-digit NDC level for each succeeding year through 2019. We used the median unit price in the first year observed to represent the baseline average manufacturer price (AMP) and inflated the drug’s baseline AMP by 2% in each subsequent year. We adjusted for the Medicaid inflation penalty by deducting any amount reimbursed above the inflation-adjusted AMP weighted by total unit fills (using the 1992-2019 files; see eMethods in the Supplement for further details regarding the inflation-based rebate calculation).^13,14^ All dollars were then adjusted to 2019 US dollars using the Consumer Price Index for All Urban Consumers. Finally, to estimate the percentage of Medicaid spending contributed by products granted accelerated approval, we summed annual gross and net Medicaid spending between 2015 and 2019 for all drugs, applying annual rebate amounts reported by the Medicaid and CHIP Payment and Access Commission (MACPAC), which includes inflation-based adjustments.

### Statistical Analysis

We calculated the percentage of total net spending on all drugs that state Medicaid programs spent on drugs with accelerated approval each year. In addition, for each year, we determined the percentage of the total number of prescriptions that were for drugs with accelerated approval. We also identified the top 10 drugs with accelerated approval by net Medicaid spending in 2019, along with the net average spending per year per product starting in the first full calendar year after a product’s approval. All analyses were conducted in Stata, version 16 (StataCorp). See eMethods in the Supplement for further details on external validation of these analyses.

### Sensitivity Analysis

We identified 19 drugs with accelerated approval that remained in the study sample, that had at least 1 indication that did not have accelerated approval, and whose indication-specific relative market shares were indeterminable. Because indication is not a factor in how prescription drugs are billed to Medicaid, inclusion of these products might overestimate spending on products with accelerated approval. Therefore, we repeated the previously described analysis after excluding these 19 drugs.

## Results

After examining the biannual CDER reports since the inception of the accelerated approval pathway in 1992 through the end of 2020, a total of 216 product-indication pairs were identified and included in this analysis (see eTable 2 in the Supplement for a complete list), comprising 149 unique products. In the pathway’s first decade (1992-2001), of 40 product-indication pairs with accelerated approval, 16 (40.0%) were targeted for HIV/AIDS vs 12 (30.0)% for cancer and 12 (30.0%) for other conditions (Figure). Over time, products with accelerated approval that were indicated for cancer subtypes have increased, representing 28 of 30 (93.3%) product-indication pairs approved in 2020. The number of product-indication pairs receiving accelerated approval status has also increased over time, with the mean annual number of product-indication pairs approved in the most recent decade (12.9 approvals in 2011-2020) being nearly 3 times the annual average over the first 2 decades of the program (4.6 approvals in 1992-2010).

**Figure.**
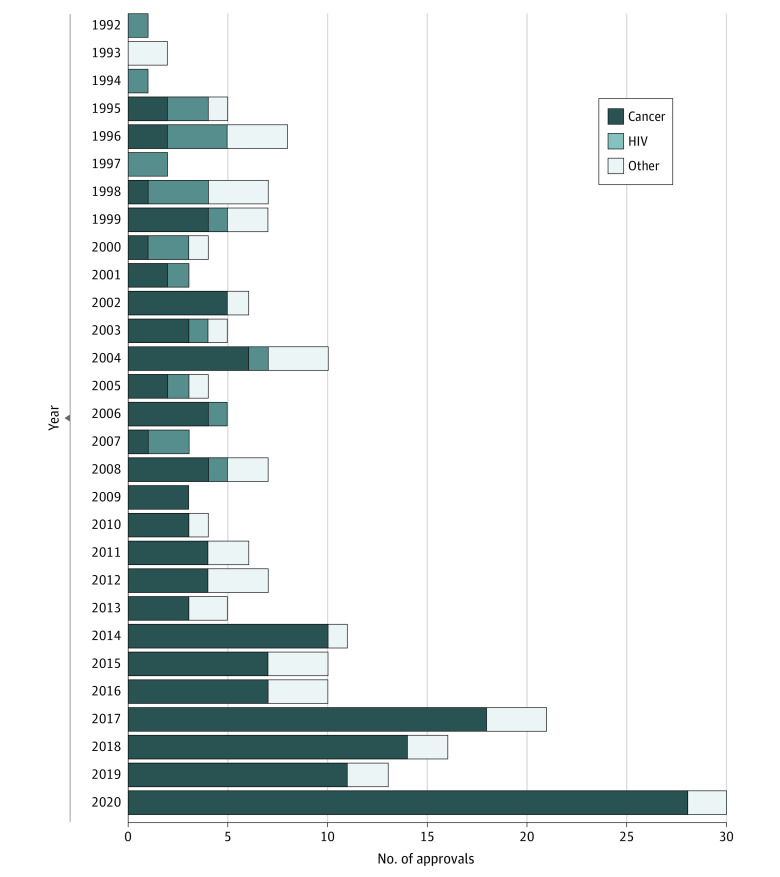
Products and Associated Indications Approved Through the US Food and Drug Administration’s Accelerated Approval Program, 1992-2020

### Medicaid Use and Spending on Products With Accelerated Approval

There were 183 product-indication pairs with accelerated approval, consisting of 121 unique products reimbursed by Medicaid between 2015 and 2019 (eFigure and eTable 2 in the Supplement). Relative to all drugs paid for by Medicaid programs, between 2015 and 2019, products with accelerated approval as a percentage of total outpatient prescription use comprised between 0.2% and 0.4% of use, or 1.3 million to 2.4 million prescriptions annually, declining each year after 2015 (Table 2).

**Table 2.** Spending and Use of Drugs With Accelerated Approval Relative to All Drugs^a^

Year	Drugs with accelerated approval			All drugs			Spending on drugs with accelerated approval, %	Prescriptions of drugs with accelerated approval, %
	Gross spending, $	Net spending, $	Prescriptions	Gross spending, $	Net spending, $	Prescriptions		
2015	4 156 129 280	2 205 562 624	2 418 753	62 803 562 496	34 604 761 088	696 103 872	6.4	0.4
2016	4 652 082 176	2 482 836 224	2 285 451	69 404 336 128	33 869 318 144	744 184 320	7.3	0.3
2017	4 861 428 736	2 601 984 512	1 998 629	71 650 836 480	32 601 131 008	764 512 704	8.0	0.3
2018	4 696 761 344	2 508 952 320	1 550 386	68 267 962 368	27 648 522 240	740 410 496	9.1	0.2
2019	4 691 189 760	2 592 940 288	1 304 768	68 526 755 840	30 357 352 448	699 248 064	8.5	0.2

^a^ Gross spending represents the total amount reported in the State Medicaid Drug Utilization Data files reimbursed by Medicaid for the drugs in question, inclusive of both federal and state shares. Net spending reduces this total to account for Medicaid rebates paid back by manufacturers under the program. These figures are expected to be higher than the actually reimbursed amounts, which are based on average manufacturer price data that are not publicly available.

Gross annual spending on drugs with accelerated approval ranged from approximately $4.2 billion to $4.9 billion (in 2015 and 2017, respectively), while annual gross spending for all drugs (including drugs with accelerated approval) ranged from approximately $62.8 billion to $71.7 billion (in 2015 and 2017, respectively) (Table 2). Statutory and inflation-based rebates reduced this estimated gross spending by 46.3% for drugs with accelerated approval and by 53.2% for all drugs, such that in 2015, net spending on drugs with accelerated approval was $2.2 billion, which has increased over time to an estimated $2.6 billion in 2019 (Table 2). Despite their low use rates, annual net spending on drugs with accelerated approval represented 6.4% to 9.1% of net spending on all drugs covered by Medicaid, ranging from $27.6 billion to $34.6 billion over the study period (Table 2).

### Top 10 Drugs With Accelerated Approval

Among the top 10 drugs with accelerated approval in 2019 by net spending are products with diverse indications, including pembrolizumab ($155.0 million) to treat numerous cancers, tenofovir disoproxil fumarate and emtricitabine ($147.8 million) for the treatment of HIV infection, hydroxyprogesterone caproate injection ($124.3 million) to reduce the risk of recurrent preterm birth, and eteplirsen ($123.9 million) to treat Duchenne muscular dystrophy (Table 3). Statutory and inflation-based rebates dramatically reduced spending for many of the top 10 drugs with accelerated approval, most notably for tenofovir disoproxil fumarate and emtricitabine (estimated rebate of 67.5% from $455 million gross spending) and pembrolizumab (estimated rebate of 49.8% from $308.6 million gross spending).

**Table 3.** Top 10 Drugs With Accelerated Approval by Net Spending in 2019

Proprietary name	Active ingredient(s)	First indication with accelerated approval	Approval year	Total reimbursement, $		
				Gross (2019)	Net (2019)^a^	Net average/y since approval^b^
Truvada	Tenofovir disoproxil fumarate and emtricitabine	For the treatment of HIV infection	2004	455 007 840	147 818 848	224 187 550
Avastin	Bevacizumab	For the treatment of metastatic *ERBB2 *(formerly *HER2*)-negative breast cancer in combination with paclitaxel	2008	207 338 080	159 442 992	155 474 551
Makena	Hydroxyprogesterone caproate injection	To reduce the risk of preterm birth in women with a singleton pregnancy and a history of singleton spontaneous preterm birth	2011	161 694 160	124 342 816	118 826 324
Perjeta	Pertuzumab	Neoadjuvant treatment in combination with trastuzumab and docetaxel for patients with *ERBB2 *(formerly *HER2*)-positive breast cancer	2013	121 797 720	93 662 448	69 591 178
Imbruvica	Ibrutinib	For the treatment of mantle cell lymphoma	2013	110 119 488	76 098 624	38 511 955
Opdivo	Nivolumab	For the treatment of unresectable or metastatic melanoma and disease progression following ipilimumab therapy	2014	215 100 512	165 412 304	123 009 929
Keytruda	Pembrolizumab	For the treatment of unresectable or metastatic melanoma and disease progression following ipilimumab therapy	2014	308 625 920	155 043 200	66 731 640
Ibrance	Palbociclib	For the treatment of postmenopausal women with estrogen receptor–positive, *ERBB2 *(formerly *HER2*)-negative advanced breast cancer in combination with letrozole	2015	302 688 128	210 968 672	180 525 260
Jadenu	Deferasirox	For the treatment of chronic iron overload in patients ≥10 y with nontransfusion-dependent thalassemia syndromes	2015	166 882 992	85 344 304	97 071 340
Exondys 51	Eteplirsen	For the treatment of Duchenne muscular dystrophy in patients who have a gene alteration amenable to exon 51 skipping	2016	161 106 800	123 891 136	74 338 275

^a^ Net spending for each product is the sum of its National Drug Code total reimbursement amount after deducting a minimum rebate (23.1% in 2019) and any inflation-based rebates estimated from a trend line beginning in the first year each National Drug Code is observed.

^b^ Beginning in the first full calendar years of sales, net average per year is the average of the annual Consumer Price Index for All Urban Consumers–adjusted (2019 US dollars) net spending for each product. This figure may be greater than net spending in 2019 owing to larger volumes of sales or higher prices for a given drug in preceding years.

### Sensitivity Analysis

Annual use for drugs with accelerated approval decreased by 9.3% (approximately 225 000 prescriptions) to 24.9% (approximately 325 000 prescriptions), and net spending decreased by 18.1% ($400 million) to 19.7% ($493 million) after excluding the 19 products both with and without indications that received accelerated approval (eTable 3 in the Supplement). Despite this, estimates of spending and use on products with accelerated approval as a proportion of all drug spending and use were similar to the primary analysis. Specifically, after excluding these products, drugs with accelerated approval made up 5.3% to 7.4% of total net spending (from $1.8 billion to $2.1 billion) and 0.1% to 0.3% of outpatient prescription drug use (from approximately 980 000 to 2.2 million prescriptions) (see eTable 3 in the Supplement for full analysis).

## Discussion

These findings provide evidence that the drugs approved through the FDA’s growing accelerated approval program represent a disproportionate share of overall Medicaid drug spending relative to their small percentage of Medicaid use. Prior studies have demonstrated that drugs costing more than $1000 per claim, which is the case for many drugs with accelerated approval, made up just 1.2% of all prescription drug claims in Medicaid but 43.7% of total drug spending.^15^ These high-cost drugs have already been identified as an area of concern for state prescription drug spending, but the broad range of products with high prices has made it difficult for states and policy makers to identify tractable policy solutions. The present findings highlight a subset of high-cost drugs for which legal and policy tools may be available to manage spending growth.

The magnitude and share of state Medicaid spending for drugs with accelerated approval is one important piece of evidence supporting states’ concerns with the accelerated approval program. States’ expressed concerns often go a step further, however, stating that the surrogate end point relied on by the sponsor and the FDA to demonstrate clinical efficacy may not be predictive of the true clinical end point. As summarized in Table 3, hydroxyprogesterone caproate injection would be one such example: its 2011 approval was based on its apparent ability to reduce the risk of recurrent preterm birth,^16^ as a surrogate for improved neonatal outcomes. In 2019, however, the required confirmatory trial failed to reproduce these findings on the surrogate end point of preterm birth or to improve neonatal outcomes.^9^ In October 2020, the FDA proposed withdrawing hydroxyprogesterone caproate injection’s approval,^9^ as is permitted by the accelerated approval statute, but the drug’s sponsor has not agreed and is seeking an agency hearing.^17^ Between 2012 and 2019, state Medicaid programs spent on average an estimated $118 826 324 per year on branded hydroxyprogesterone caproate injection (net of rebates and inflation penalties) while seemingly achieving little or no therapeutic benefit for patients.

The present analysis regarding overall Medicaid spending on these products is also important even for those products that have completed their follow-on clinical trial requirements. Previous analyses have demonstrated that many oncology drugs receiving accelerated approval analyze either the same or a different surrogate end point in their confirmatory trials, rather than establishing a real clinical benefit.^8^ As a result, states’ concerns regarding the clinical value of their spending on drugs with accelerated approval are unlikely to be limited to those that have not yet completed their confirmatory trials. The present findings help illuminate the scope of potential policy interventions such as 2 recent April 2021 proposals from expert bodies. The MACPAC voted to recommend changing the terms on which Medicaid programs pay for drugs with accelerated approval.^11^ The MACPAC proposed that Congress take steps to increase the mandatory Medicaid rebates (higher than the currently required 23.1%) for products with accelerated approval, either beginning at approval and continuing until manufacturers have verified their products’ clinical benefits or going into effect a specified number of years after approval if the manufacturer has not yet completed the required postmarketing trials. Either of these approaches could have resulted in considerable state savings on hydroxyprogesterone caproate injection, among other products. And the Institute for Clinical and Economic Review published a white paper proposing a range of policy reforms to the accelerated approval program, including several that would focus on Medicaid, including increasing mandatory minimum rebates and creating outcomes-based contracts.^12^

More generally, as noted in the Figure, nearly all products approved through the accelerated approval program in recent years are either for oncology or rare-disease indications. This represents a shift from the program’s initial origins as a tool to speed HIV/AIDS medications to market. Any policy interventions would be more likely to affect those disease classes rather than HIV/AIDS medications.

### Limitations

This study has several limitations that deserve discussion. First, CMS’s Medicaid expenditure database is incomplete in ways that may lead the present analysis to underestimate program spending and use related to products with accelerated approval. For instance, the database does not include drugs when used in the inpatient setting or in the context of the 340B drug pricing program. Furthermore, the present data include NDCs only for brand-name products and do not capture spending on and use of generic or biosimilar forms of the older drugs in the analysis.

Second, CMS’s Medicaid expenditure database does not include information on spending by indication, reporting only spending per drug compound. To be conservative in our estimates, we excluded 5 drugs with accelerated approval where we expected high overall use for the product but relatively rare use for the indication that received accelerated approval. We also completed sensitivity analyses excluding an additional 19 drugs that had any indications that did not receive accelerated approval to provide a lower bound for our spending and use estimates.

Third, given the nonpublic nature of both AMP reporting and drug-specific rebates, the analysis provides estimated figures for spending totals. We approximate AMP using the median unit price reported in the Medicaid spending data and adjust for rebates, yet the estimated gross- and net-spending totals are higher than those reported by the MACPAC in the aggregate, for reasons we explore in eMethods and eTable 4 in the Supplement. To determine rebate levels for drugs with accelerated approval, we applied the mandatory minimum Medicaid rebate of 23.1% because products with accelerated approval are unlikely to face price competition, meaning that states will find it difficult to negotiate additional, supplemental rebates. By definition, the accelerated approval program is designed for drugs that may fulfill unmet medical needs, and many drugs with accelerated approval are first-in-class products and cancer drugs for which rebates are typically low. We estimated product-specific inflation-based rebates using annual median prices for filled products across managed care and fee-for-service contexts and summarized at the national level. Importantly, the trends and proportions we report are consistent with previous research.

## Conclusions

This cross-sectional study demonstrates that state spending on products with accelerated approval represents an outsized amount of spending relative to their use. States are understandably concerned that the limited evidence of efficacy present at approval in some of these cases will not hold up on further review and that as a result they are devoting considerable amounts of spending to products with unproven clinical benefits. Although we give examples herein of particular drugs where that appears to be the case, a fuller accounting of state spending attributable to these products will require further study.

## Notes

Supplement. eMethods. eFigure. Flow Diagram: Accelerated Approval Drugs, 1992-2020 eTable 1. Accelerated Approval Product-Indications Excluded from Primary Analysis eTable 2. Accelerated Approval Product-Indication Pairs Study Sample in Primary Analysis eTable 3. Sensitivity Analysis of Spending and Utilization of Drugs With Accelerated Approval Excluding Drugs With Non–Accelerated Approval and Accelerated Approval Indications eTable 4. “Non-Medicaid” Amount Reimbursed as a Percentage of Total Amount Reimbursed
