# Supplementary material for: Recent Trends in Medicaid Spending and Use of Drugs With US Food and Drug Administration Accelerated Approval
Source: JAMA Health Forum. 2021 Oct 8;2(10):e213177. doi: 10.1001/jamahealthforum.2021.3177 (PMC8727037; doi:10.1001/jamahealthforum.2021.3177)
Supplement: Supplement. — eMethods. eFigure. Flow Diagram: Accelerated Approval Drugs, 1992-2020 eTable 1. Accelerated Approval Product-Indications Excluded from Primary Analysis eTable 2. Accelerated Approval Product-Indication Pairs Study Sample in Primary Analysis eTable 3. Sensitivity Analysis of Spending and Utilization of Drugs With Accelerated Approval Excluding Drugs With Non–Accelerated Approval and Accelerated Approval Indications eTable 4. “Non-Medicaid” Amount Reimbursed as a Percentage of Total Amount Reimbursed [file jamahealthforum-e213177-s001.pdf]

Supplemental Online Content

Sachs RE, Gavulic KA, Donohue JM, Dusetzina SB. Recent trends in Medicaid spending and use of drugs with US Food and Drug Administration accelerated approval. *JAMA Health Forum*. 2021;2(10):e213177. doi:10.1001/jamahealthforum.2021.3177

eMethods.

eFigure. Flow Diagram: Accelerated Approval Drugs, 1992-2020

eTable 1. Accelerated Approval Product-Indications Excluded from Primary Analysis

eTable 2. Accelerated Approval Product-Indication Pairs Study Sample in Primary Analysis

eTable 3. Sensitivity Analysis of Spending and Utilization of Drugs With Accelerated Approval Excluding Drugs With Non–Accelerated Approval and Accelerated Approval Indications

eTable 4. “Non-Medicaid” Amount Reimbursed as a Percentage of Total Amount Reimbursed

This supplemental material has been provided by the authors to give readers additional information about their work.

© 2021 Sachs RE et al. *JAMA Health Forum.*

I. eMethods

Details: Creation of study sample

We constructed a data set at the product-indication level of all AA products approved by the FDA between December 1992-2020, using a biannual report from the FDA's CDER. We cross-checked each approval to CDER's compilation of all new molecular entities and biological approvals from 1985-2019 to identify any products that had a previously approved indication under the traditional approval pathway (i.e. "non-AA indication"). We elected not to include any AA products that were approved by the Center for Biologics Evaluation and Research (CBER), as we determined public access to these data to be unreliable.

We completed a crosswalk on brand names for the full AA list to match each drug to 11-digit NDC codes, using Redbook. When not found in Redbook, we used a publicly available search tool (e.g. ATLAS) to append additional NDC-11 codes. We matched the NDC-11 codes to our cleaned AA list and linked this file to spending and utilization data found at Medicaid.gov. For each year between 1992-2019, we downloaded the "Full Dataset (States + Totals)" and converted each file to a Stata .dta file. To perform our analyses more efficiently, we output a compressed data file that grouped "Total amount reimbursed," "Medicaid amount reimbursed," "non-Medicaid amount reimbursed," "number of units reimbursed," and "number of prescriptions reimbursed" by accelerated approval status, state, year, quarter, and utilization type. For AA drugs, we further grouped by prefix product name and NDC-11 code.

Details: Calculations for inflation-based rebates

To account for the Medicaid inflation penalty, we identified the first year each NDC-11 code was observed between 1992-2019 and estimated the median unit price at the NDC-11 code-level for each succeeding year through 2019. We used the median unit price in that year to represent the baseline average manufacturer price (AMP). For each subsequent year, we inflated the drug's baseline AMP by 2%, calculating a target price in each year from 2015 to 2019. The statutory inflation-based rebated for each NDC-11 code was then estimated as any unit price increase exceeding the 2% trend line from its baseline AMP multiplied by the NDC-11 code's total unit fills. This method enabled us to take into account both price and volume of sales in any rebate adjustment due to inflation. Each product's total annual inflation-based rebate was the sum of inflation-based rebates for each of its NDC-11 codes.

To finalize this method described above, we performed numerous variations to select the parameters that would best estimate each drug's AMP and our ability to account for year-to-year changes in price since market entry. Applying inflation-based rebates at the NDC-11 code-level required using a measure of central tendency to summarize unit prices across quarters in a given year and across utilization context. For this step, we tested using both the mean and the median. After closely assessing unit prices in the 25th and 75th percentiles among select AA drugs relative to their mean and median unit prices, we selected to estimate inflation-based rebates on the median unit price to safeguard against any arithmetic bias introduced by outliers. We also carefully considered the year that would represent a NDC-11 code's "baseline AMP," important for estimating inflation-based rebates that would accurately account for changes in price and volume of sales since a drug's market entry. It was critical to begin each NDC-11 code's inflation-adjusted trendline in the first year that is observed in the data, which often immediately follows FDA approval. Finally, since the State Medicaid Drug Utilization files are not indication specific, we used the first year each NDC-11 code was observed between 1992-2019 as the baseline year, even if the first year observed came before the AA indication approval date.

Details: External validation

To validate our findings, we compared our estimated gross and net Medicaid spending amounts to the available MACStats: Medicaid and CHIP Data Books, which include aggregated total gross

© 2021 Sachs RE et al. *JAMA Health Forum.*

Medicaid spending and rebate amounts for years 2015-2018. We found our gross and net estimates in each year to be slightly higher, which can be accounted for by several factors. First, in contrast to MACPAC, we relied on the field for “total amount reimbursed” in the State Medicaid Drug Utilization files, as opposed to “Medicaid amount reimbursed” alone. Total spending includes a small percentage of non-Medicaid spending for AA drugs (<10%) and for all outpatient prescription drugs in any given year (<7%), as shown in eTable 4. While we would have preferred to calculate total gross and net Medicaid spending using the “Medicaid amount reimbursed,” the fields for “Medicaid amount reimbursed” and “non-Medicaid amount reimbursed” did not consistently sum to “total amount reimbursed” prior to 2008. Our use of “total amount reimbursed” was thus important to avoid largely inaccurate baseline AMP estimates for AA drugs entering the market prior to 2008, which would have important consequences for estimating inflation-based rebates during each year of our study period. In addition, the source of non-Medicaid spending, defined by CMS as “the amount reimbursed by non-Medicaid entities to pharmacies or other providers for the 11-digit NDC FFS or MCO drug in the quarter/year covered…[and] includes any drug reimbursement amount for which the state is not eligible for federal matching funds” was unclear. Second, our analysis is based upon calendar year versus fiscal year, which MACPAC reports on. Third, whereas we directly compute rebate amounts, MACPAC relies upon the CMS-64 FMR net expenditure data files that report rebate dollars that do not necessarily align with a given study period.

© 2021 Sachs RE et al. *JAMA Health Forum.*

II. Tables and Figures

eFigure. Flow Diagram: Accelerated Approval Drugs, 1992-2020

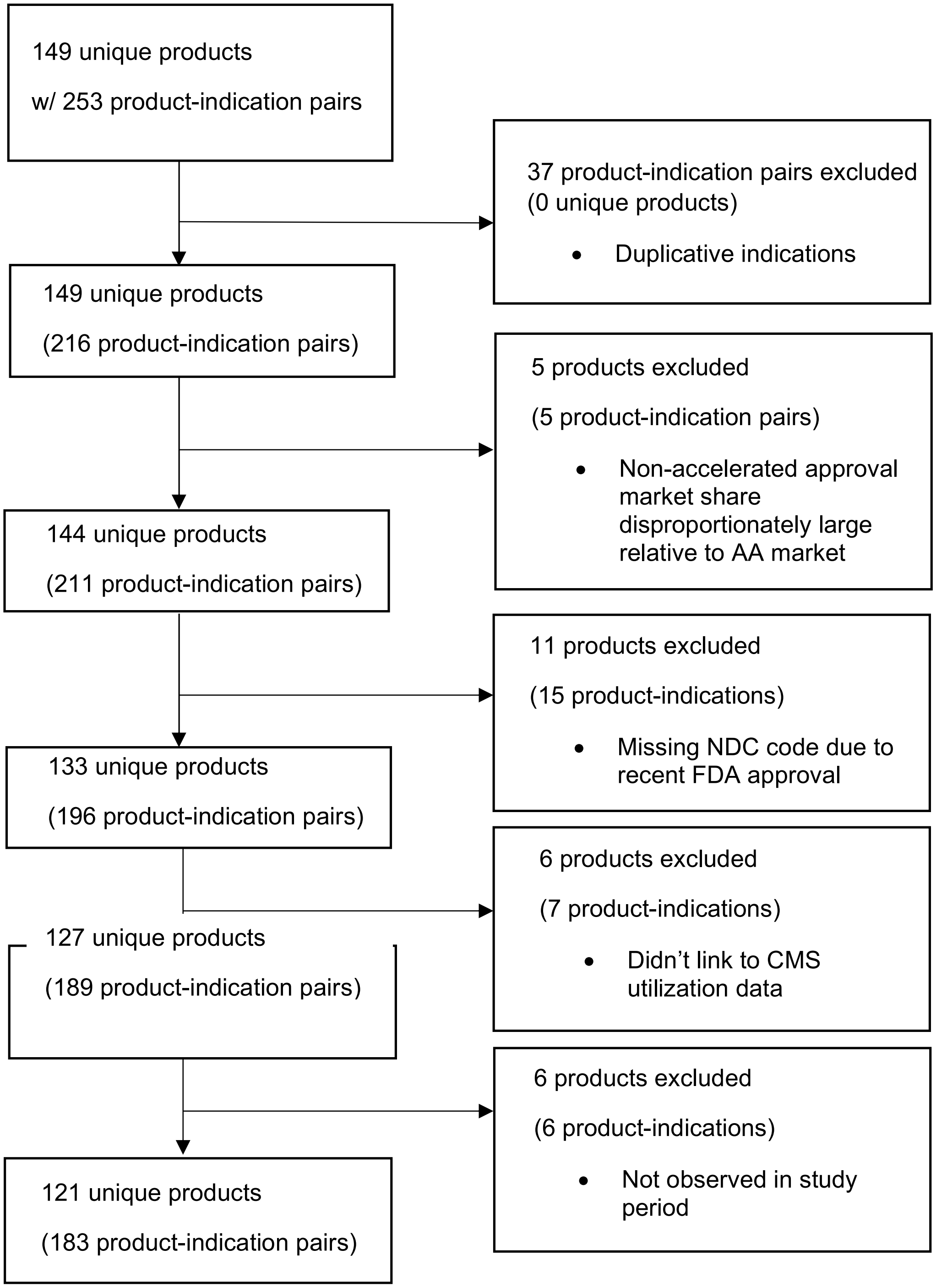

© 2021 Sachs RE et al. *JAMA Health Forum.*

eTable 1. Accelerated Approval Product-Indications Excluded from Primary Analysis						
**Proprietary Name**	**Active Ingredient**	**FDA Receipt Date**	**FDA Approval Date**	**Supplement**	**Accelerated Approval Indication**	**Exclusion Category**
Hivid	zalcitabine	10/31/1991	6/19/1992	No	in combination with zidovudine as indicationed for the treatment of adult patients with advanced hiv infection (cd4 cell counts < 300 cells/mm3) who have demonstrated significant clinical or immunologic deterioration	Not observed in study period
Biaxin	clarithromycin	11/2/1992	12/23/1993	No	for the treatment of disseminated mycobacterial infections due to mycobacterium avium and mycobacterium intracellulare	Duplicate indication
Biaxin	clarithromycin	11/2/1992	12/23/1993	No	for the treatment of disseminated mycobacterial infections due to mycobacterium avium and mycobacterium intracellulare	Large non-AA market share relative to AA share
Epivir	lamivudine	7/7/1995	11/17/1995	No	in combination with retrovir (zidovudine) for the treatment of hiv infection when therapy is warranted based on clinical and/or immunological evidence of disease progression	Duplicate indication
Norvir	ritonavir	12/21/1995	3/1/1996	No	in combination with nucleoside analogs or as monotherpy for the treatment of hiv infection when therapy is warranted	Duplicate indication
ProAmatine	midodrine hydrochloride	9/25/1995	9/6/1996	No	for the treatment of sympotomatic orthostatic hyprotension (oh)	Not observed in study period

© 2021 Sachs RE et al. *JAMA Health Forum.*

Viracept	nelfinavir mesylate	12/26/1996	3/14/1997	No	for the treatment of hiv infection when therapy is warranted	New forumulation
Viramune	nevirapine	3/16/1998	9/11/1998	Yes	provides for an oral suspension in combination with other antiretroviral agents for the treatment of hiv-1 infection	New forumulation
Ziagen	abacavir sulfate	6/24/1998	12/17/1998	No	in combination with other antiretroviral agents, for the treatment of hiv-1 infection	New formulation
Ontak	denileukin diftitox	12/9/1997	2/5/1999	No	for the treatment of persistent or recurrent cutaneous t-cell lymphoma whose malignant cells express the cd25 component of the il-2 receptor	Not observed in study period
Agenerase	amprenavir	10/16/1998	4/15/1999	No	in combination with other antiretroviral agents, for the treatment of hiv-1 infection	New formulation
Agenerase	amprenavir	12/8/1998	4/15/1999	No	in combination with other antiretroviral agents, for the treatment of hiv-1 infection	Not observed in study period
Synercid	dalfopristin, quinupristin	9/5/1997	9/21/1999	No	for the treatment of vancomycin-resistant enterococcus faecium (vref)	Large non-AA market share relative to AA share
Celebrex	celecoxib	6/25/1999	12/23/1999	No	to reduce the number of adenomatous colorectal polyps in familial adenomatous polyposis patients, as an adjunct to ususal care	Large non-AA market share relative to AA share
Cipro	ciprofloxacin	3/2/2000	8/30/2000	Yes	for the treatment of inhalational anthrax (post-exposure)	Duplicate indication
Cipro	ciprofloxacin	3/2/2000	8/30/2000	Yes	for the treatment of inhalational anthrax (post-exposure)	Duplicate indication
Cipro	ciprofloxacin	3/2/2000	8/30/2000	Yes	for the treatment of inhalational anthrax (post-exposure)	Duplicate indication

© 2021 Sachs RE et al. *JAMA Health Forum.*

Cipro	ciprofloxacin	3/1/2000	8/30/2000	Yes	for the treatment of inhalational anthrax (post-exposure)	Duplicate indication
Cipro	ciprofloxacin	3/2/2000	8/30/2000	Yes	for the treatment of inhalational anthrax (post-exposure)	Large non-AA market share relative to AA share
Kaletra	lopinavir, ritonavir	6/1/2000	9/15/2000	No	in combination with other antiretroviral agents for the treatment of hiv-1 infection in adults and pediatric patients age six months or older	New formulation
Luveris	lutropin alpha	5/1/2001	10/8/2004	No	concomitantly adminiistered with gonal-f (follitropin alfa for injection) for stimulation of follicular development in infertile hypogonadotropic hypogonadal women with profound lh deficiency(lh < 1.2 iu/l)	Didn't link to utilization data
Remodulin	treprostinil sodium	1/30/2004	11/24/2004	Yes	provides for adding the infusion of remodulin (treprostinil sodium) 1, 2.5, 5 & 10 mg/ml injection via an indwelling central venous catheter to the labeling for the treatment of pulmonary arterial hypertension (pah)	New formulation
Levaquin	levofloxacin	5/26/2004	11/24/2004	Yes	for the treatment of inhalational anthrax (post-exposure)	Duplicate indication
Levaquin	levofloxacin	5/26/2004	11/24/2004	Yes	for the treatment of inhalational anthrax (post-exposure)	Duplicate indication
Bexxar	tositumomab, iodine I 131 tositumomab	7/3/2004	12/22/2004	Yes	expand the indication to include patients with relapsed or refractory, low grade, follicular or transformed cd20 positive non-hodgkin's lymphoma who have not received rituximab	Not observed in study period

© 2021 Sachs RE et al. *JAMA Health Forum.*

Gleevec	imatinib mesylate	3/28/2006	9/27/2006	Yes	for the treatment of newly diagnosed philadelphia positive cml in pediatric patients	Extension to pediatric population
Levaquin	levofloxacin	7/5/2007	5/5/2008	Yes	to reduce the incidence or progression of disease following exposure to aerosolized bacillus anthracis in pediatric patients (>6 month of age and older)	Duplicate indication
Levaquin	levofloxacin	7/5/2007	5/5/2008	Yes	to reduce the incidence or progression of disease following exposure to aerosolized bacillus anthracis in pediatric patients (>6 month of age and older)	Duplicate indication
Oforta	fludarabine phosphate	11/29/2007	12/18/2008	No	for the treatment of adults pts with b cell chronic lymphocytic leuemia (cll) whose disease has not responded to or who have not responded to or has progressed during or after treatment with at least one standard alkylating-agent containing regimen	Not observed in study period
Elaprase	idursulfase	9/24/2012	6/24/2013	Yes	provides for additional safety and efficacy information for the treatment of patients with hunter syndrome 5 years of age and younger	Large non-AA market share relative to AA share
Ferriprox	deferiprone	11/17/2014	9/9/2015	No	for the treatment of patients with transfusional iron overload due to thalassemia syndromes when current chelation therapy is inadequate	Duplicate indication

© 2021 Sachs RE et al. *JAMA Health Forum.*

Imbruvica	ibrutinib	8/31/2017	2/16/2018	No	for the treatment of adult patients with marginal zone lymphoma (mzl) who require systemic therapy and have received at least one prior anti- cd20 based therapy	Duplicate indication
Vitrakvi	larotrectinib	3/26/2018	11/26/2018	No	for the treatment of adult and pediatric patients with solid tumors that have a neurotrophic receptor tyrosine kinase (ntrk) gene fusion without a known acquired resistance mutation; are metastatic or where surgical resection is likely to result in severe morbidity; and have no satisfactory alternative treatments or that have progressed following treatment	New formulation
Sirturo	bedaquiline	10/11/2018	8/9/2019	Yes	as part of combination therapy for the treatment of adult and pediatric patients (12 to less than 18 years of age and weighing at least 30kg) with pulmonary multi-drug resistant tuberculosis (mdr-tb)	Extension to pediatric population
Brukinsa	zanubrutinib	6/27/2019	11/14/2019	No	for the treatment of adult patients with mantle cell lymphoma (mcl) who have received at least one prior therapy	Didn't link to utilization data
Vyondys 53	golodirsen	12/19/2018	12/12/2019	No	for the treatment of duchenne muscular dystrophy (dmd) in patients who have a confirmed mutation of the dmd gene that is amenable to exon 53 skipping	Didn't link to utilization data

© 2021 Sachs RE et al. *JAMA Health Forum.*

Padcev	enfortumab vedotin-ejfv	7/15/2019	12/18/2019	No	for the treatment of adult patients with locally advanced or metastatic urothelial cancer (muc) who have previously received a programmed death receptor-1 (pd-1) or programmed death-ligand 1 (pd-l1) inhibitor, and a platinum-containing chemotherapy in the neoadjuvant/adjuvant, locally advanced or metastatic setting	Didn't link to utilization data
Enhertu	fam-trastuzumab deruxtecan-nxki	8/29/2019	12/20/2019	No	for the treatment of adult patients with unresectable or metastatic her2-positive breast cancer who have received two or more prior anti-her2-based regimens in the metastatic setting	Didn't link to utilization data
Tazverik	tazemetostat	5/23/2019	1/23/2020	No	for the treatment of adults and pediatric patients aged 16 years and older with metastatic or locally advanced epithelioid sarcoma not eligible for complete resection	Didn't link to utilization data
Romidepsin	romidepsin	8/18/2015	3/13/2020	No	treatment of peripheral t-cell lymphoma (ptcl) in adult patients who have received at least one prior therapy	Duplicate indication
Pemazyre	pemigatinib	9/30/2019	4/17/2020	No	for the treatment of adults with previously treated, unresectable locally advanced or metastatic cholangiocarcinoma with a fibroblast growth factor receptor 2 (fgfr2) fusion or other rearrangement as	Missing NDC code

© 2021 Sachs RE et al. *JAMA Health Forum.*

					detected<br>by an fda-approved test	
Trodelvy	sacituzumab	5/18/2018	4/22/2020	No	for the treatment of adult patients with metastatic triple-negative breast cancer (mtnbc) who have received at least two prior therapies for metastatic disease	Missing NDC code
Keytruda	pembrolizumab	4/20/2020	4/28/2020	Yes	provides for an alternate dose/schedule of 400mg every 6 weeks for adult patients with unresectable or metastatic microsatellite instability-high (msi-h) or mismatch repair deficient (dmmr) solid tumors that have progressed following prior tx and who have no satisfactory alternative tx options	Duplicate indication
Keytruda	pembrolizumab	4/15/2020	4/28/2020	Yes	provides for an alternate dose/schedule of 400mg every 6 weeks for adult patients with metastatic sclc with disease progression on or after platinum-based chemotherapy and at least one other line of therapy	Duplicate indication
Keytruda	pembrolizumab	4/14/2020	4/28/2020	Yes	provides for an alternate dose/schedule of 400mg every 6 weeks for adult patients with advanced cervical cancer with disease progression during or	Duplicate indication

© 2021 Sachs RE et al. *JAMA Health Forum.*

					following chemotherapy	
Keytruda	pembrolizumab	4/14/2020	4/28/2020	Yes	provides for an alternate dose/schedule of 400mg every 6 weeks for adult patients with endometrial cancer	Duplicate indication
Keytruda	pembrolizumab	9/23/2019	4/28/2020	Yes	provides for an alternate dose/schedule of 400mg every 6 weeks for adult patients with non-small cell lung cancer (nsclc)	Duplicate indication
Keytruda	pembrolizumab	4/23/2019	4/28/2020	Yes	provides for an alternate dose/schedule of 400mg every 6 weeks for adult patients with advanced or metastatic gastric or gastroesophageal junction adenocarcinoma	Duplicate indication
Keytruda	pembrolizumab	4/23/2019	4/28/2020	Yes	provides for an alternate dose/schedule of 400mg every 6 weeks for adult patients with merkel cell carcinoma	Duplicate indication
Keytruda	pembrolizumab	4/23/2019	4/28/2020	Yes	provides for an alternate dose/schedule of 400mg every 6 weeks for adult patients with hepatocellular carcinoma (hcc) who have been previously treated with an antiangiogenic tyrosine kinase inhibitor (tki)	Duplicate indication

© 2021 Sachs RE et al. *JAMA Health Forum.*

Keytruda	pembrolizumab	4/23/2019	4/28/2020	Yes	provides for an alternate dose/schedule of 400mg every 6 weeks for adult patients with hematological malignancies: primary mediastinal b-cell lymphoma	Duplicate indication
Keytruda	pembrolizumab	4/23/2019	4/28/2020	Yes	provides for an alternate dose/schedule of 400mg every 6 weeks for adult patients with hematological malignancies: hodgkin	Duplicate indication
Keytruda	pembrolizumab	4/18/2019	4/28/2020	Yes	provides for an alternate dose/schedule of 400mg every 6 weeks for adult patients with unresectable or metastatic melanoma	Duplicate indication
Tabrecta	capmatinib	12/10/2019	5/6/2020	No	for the treatment of adult patients with metastatic non-small cell lung cancer (nsclc) whose tumors have a mutation that leads to mesenchymal-epithelial transition (met) exon 14 skipping as detected by an fda-approved	Missing NDC code
Retevmo	selpercatinib	12/4/2019	5/8/2020	No	for the treatment of adult and pediatric patients 12 years of age and older with advanced or metastatic ret-mutant medullary thyroid cancer (mtc) who require systemic therapy	Missing NDC code
Retevmo	selpercatinib	12/4/2019	5/8/2020	No	for the treatment of adult patients with metastatic ret fusion-positive non-small cell lung cancer (nsclc)	Missing NDC code

© 2021 Sachs RE et al. *JAMA Health Forum.*

Retevmo	selpercatinib	12/4/2019	5/8/2020	No	for the treatment of adult and pediatric patients 12 years of age and older with advanced or metastatic ret fusion-positive thyroid cancer who require systemic therapy and who are radioactive iodine-refractory (if radioactive iodine is appropriate)	Missing NDC code
Ferriprox	deferiprone	7/19/2019	5/19/2020	No	for the treatment of patients with transfusional iron overload due to thalassemia syndromes when current chelation therapy is inadequate	Duplicate indication
Sirturo	bedaquiline	11/27/2019	5/27/2020	Yes	for the treatment of pulmonary multi-drug resistant tuberculosis as part of combination therapy, in adult and pediatric patients (12 to less than 18 years of age and weighing at least 30 kg) to include patients ≥ 5 to <12 years of age and weighing at least 15 kg	Extension to pediatric population
Zepzelca	lurbinectedin	12/16/2019	6/15/2020	No	for the treatment of adult patients with metastatic small cell lung cancer (sclc) with disease progression on or after prior platinum-based chemotherapy	Missing NDC code
Keytruda	pembrolizumab	6/2/2020	6/16/2020	Yes	provides for an alternate dose/schedule of 400 mg every 6 weeks for adult patients with unresectable or metastatic tmb-h [≥10 mutations/megabase (mut/mb)] solid tumors, as	Duplicate indication

© 2021 Sachs RE et al. *JAMA Health Forum.*

					determined by an fda-approved test, that have progressed following prior treatment and who have no satisfactory alternative treatment options	
Tazverik	tazemetostat	12/18/2019	6/18/2020	No	for the treatment of adult patients with relapsed or refractory (r/r) follicular lymphoma (fl) whose tumors are positive for an ezh2 mutation as detected by an fda approved test and who have received at least 2 prior systemic therapies; and the treatment of adult patients with r/r fl who have no satisfactory alternative treatment options.	Didn't link to utilization data
Monjuvi	tafasitamab-cxix	12/30/2019	7/31/2020	No	in combination with lenalidomide for the treatment of adult patients with relapsed or refractory diffuse large b-cell lymphoma (dlbcl) not otherwise specified, including dlbcl arising from low grade lymphoma, and who are not eligible for autologous stem cell transplant (asct)	Missing NDC code
Blenrep	belantamab mafodotin-blmf	12/5/2019	8/5/2020	No	for the treatment of adults with relapsed or refractory multiple myeloma who have received at least four prior therapies including an anti-cd38 monoclonal antibody, a proteasome inhibitor, and an immunomodulatory agent	Missing NDC code

© 2021 Sachs RE et al. *JAMA Health Forum.*

Lampit	nifurtimox	12/6/2019	8/6/2020	No	for the treatment of chagas disease in pediatric patients birth to less than 18 years of age and weighing at least 2.5 kg	Missing NDC code
Viltepso	viltolarsen	12/12/2019	8/12/2020	No	for the treatment of duchenne muscular dystrophy (dmd) in patients who have a confirmed mutation of the dmd gene that is amenable to exon 53 skipping	Missing NDC code
Gavreto	pralsetinib	3/23/2020	9/4/2020	No	for the treatment of adult patients with metastatic ret fusion-positive non-small cell lung cancer (nsclc) as detected by an fda approved test	Missing NDC code
Danyelza	naxitamab-gqgk	3/31/2020	11/25/2020	No	in combination with granulocyte-macrophage colony-stimulating factor (gm-csf), for the treatment of pediatric patients 1 year of age and older and adult patients with relapsed or refractory high-risk neuroblastoma in the bone or bone marrow who have demonstrated a partial response, minor response, or stable disease to prior therapy	Missing NDC code
Gavreto	pralsetinib	6/30/2020	12/1/2020	No	for the treatment of adult and pediatric patients 12 years of age and older with advanced or metastatic ret fusion-positive thyroid cancer who require systemic therapy and who are radioactive iodine-refractory (if radioactive iodine is appropriate)	Missing NDC code

© 2021 Sachs RE et al. *JAMA Health Forum.*

Gavreto	pralsetinib	6/30/2020	12/1/2020	No	for the treament of adult and pediatric patients 12 years of age and older with advanced or metastatic ret-mutant medullary thyroid cancer (mtc) who require systemic therapy	Missing NDC code

© 2021 Sachs RE et al. *JAMA Health Forum.*

eTable 2. Accelerated Approval Product-Indication Pairs Study Sample in Primary Analysis					
**Proprietary Name**	**Active Ingredient**	**FDA Receipt Date**	**FDA Approval Date**	**Supplement**	**Accelerated Approval Indication**
Betaseron	interferon beta-1b	6/18/1992	7/23/1993	No	use in ambulatory patients with relapsing-remitting multiple sclerosis to reduce the frequency of clinical exacerbations
Zerit	stavudine	12/28/1993	6/24/1994	No	for the treatment of adult patients with advanced hiv infection who are intolerant of approved therapies with proven clinical benefit or who have experienced significant clinical or immunological deterioration while receiving these therapies or for whom such therapies are contraindicated
Zinecard	dexrazoxane	8/5/1994	5/26/1995	No	for the prevention of cardiomyopathy associated with doxorubicin administration
Casodex	bicalutamide	9/14/1994	10/4/1995	No	in combination therapy with an lhrh analogue for the treatment of advanced prostate cancer
Epivir	lamivudine	7/7/1995	11/17/1995	No	in combination with retrovir (zidovudine) for the treatment of hiv infection when therapy is warranted based on clinical and/or immunological evidence of disease progression
Doxil	doxorubicin hydrochloride	9/7/1994	11/17/1995	No	for the treatment of kaposi's sarcoma in aids patients with disease that has progressed on prior combination chemotherapy or in patients who are intolerant to such therapy
Invirase	saquinavir	8/31/1995	12/6/1995	No	in combination with nucleoside analogs for the treatment of advanced hiv infection in selected patients
Norvir	ritonavir	12/21/1995	3/1/1996	No	in combination with nucleoside analogs or as monotherpy for the treatment of hiv infection when therapy is warranted
Crixivan	indinavir sulfate	1/31/1996	3/13/1996	No	for the treatment of hiv-1 infection in adults when therapy is warranted

© 2021 Sachs RE et al. *JAMA Health Forum.*

Ethyol	amifostine	2/9/1996	3/15/1996	Yes	to reduct cumulative renal toxicity associated with repeated adminstations of cisplatin in patients with non-small cell lung cancer
Taxotere	docetaxel	7/27/1994	5/14/1996	No	for the treatment of patients with locally advanced or metastatic breast carcinoma who have progressed during anthracycline-based therapy or have relapsed during anthracycline-based adjuvant therapy
Camptosar	irinotecan hydrochloride	12/28/1995	6/14/1996	No	for the treatment of metastatic carcinoma of the colon or rectum whoses diease has progressed following 5-fu-based therapyc
Viramune	nevirapine	2/23/1996	6/21/1996	No	in combination with nucleoside analogues for the treatment of hiv-1 infected adults who have expreienced clinical and/or immunological deterioration
Serostim	somatropin	9/11/1995	8/23/1996	No	for the treatment of aids wasting and cachexia
Viracept	nelfinavir mesylate	12/26/1996	3/14/1997	No	for the treatment of hiv infection when antiretroviral therapy is warranted
Rescriptor	delavirdine mesylate	7/15/1996	4/4/1997	No	for the treatment of hiv-1 infection in combination with appropriate antiretroviral agents when therpay is warranted
Xeloda	capecitabine	10/31/1997	4/30/1998	No	for the treatment of metastatic breast cancer resistant to both paciltaxel and an anthracycline-containing chemotherapy regimen or resistant to paciltaxel and for whom further anthracycline therapy ay be contraindicated
Sulfamylon	mafenide acetate	3/31/1997	6/5/1998	No	for uses as adjunctive tropical antimicrobial agent to control bacterial infection when used under moist dressings over meshed autograpfts on excised burn wounds
Priftin	rifapentine	12/22/1997	6/22/1998	No	for the treatment of pulmonary tuberculosis

© 2021 Sachs RE et al. *JAMA Health Forum.*

Remicade	infliximab	12/30/1997	8/24/1998	No	for the treatment of moderately to severely active crohn's disease for the reduction of the signs and symptoms, in patients who have an inadequate response to conventional therapies and treatment of patients with fistulizing crohn's disease for the reduction in the number of draining enterocutaneous fistulas
Viramune	nevirapine	4/20/1998	9/11/1998	No	in combination with other antiretroviral agents for treatment of hiv-1 infection
Sustiva	efavirenz	6/11/1998	9/17/1998	No	in combination with other antiretroviral agents, for the treatment of hiv-1 infection
Ziagen	abacavir sulfate	6/24/1998	12/17/1998	No	in combination with other antiretroviral agents, for the treatment of hiv-1 infection
Depocyt	cyarabine(liposomal)	10/5/1998	4/1/1999	No	for the intrathecal treatment of lymphomatous meningitis
Doxil	doxorubicin hydrochloride	12/29/1998	6/28/1999	Yes	for the treatment of metastatic carcinoma of the ovary in patients with disease that is refractory to both paciltaxel-and platinum-based chemotherapy regimens
Temodar	temozolomide	8/13/1998	8/11/1999	No	for the treatment of adult patients with refractory anaplastic astrocytoma
Mylotarg	gemtuzumab ozogamicin	10/29/1999	5/17/2000	No	for the treatment of patients with cd33 positive acute myeloid leukemia in first relapse who are 60 years of age or older and who are not considered candidates for cytotoxic chemotherapy
Kaletra	lopinavir, ritonavir	6/1/2000	9/15/2000	No	in combination with other antiretroviral agents for the treatment of hiv-1 infection in adults and pediatric patients age six months or older
Trizivir	abacavir sulfate; lamivudine; zidovudine	12/17/1999	11/14/2000	No	alone or in combination with other antiretroviral agents for the treatment of hiv-1 infection
Campath	alemtuzumab	12/23/1999	5/7/2001	No	for the treatment of b-cell chronic lymphocytic leukemia (b-cll) in patients who have been treated with alkylating

© 2021 Sachs RE et al. *JAMA Health Forum.*

					agents and who have failed fludarabine therapy
Gleevec	imatinib mesylate	2/27/2001	5/10/2001	No	for the treatment of patients with chronic myeloid leukemia(cml) in blast crisis, accelerated phase, or in chronic phase after failure of interferon-alpha therap
Viread	tenofovir disoproxil fumarate	5/1/2001	10/26/2001	No	for the treatment of hiv-1 infection in adults
Gleevec	imatinib mesylate	10/16/2001	2/1/2002	Yes	for the treatment of patients with kit (cd117) positive unresectable and/or metastatic malignant gastrointestinalstromal tumors (gist)
Zevalin	ibritumomab tiuxetan	11/1/2000	2/19/2002	No	for the treatment of patients with relapsed or refractory low-grade, follicular, or transformed b-cell non-hodgkin's lymphoma(nhl) other than those patients with rituximab refractory follicular nhl
Remodulin	treprostinil sodium	10/16/2000	5/21/2002	No	treatment of pulmonary arterial hypertension
Eloxatin	oxaliplatin	6/24/2002	8/9/2002	No	in combination with infusional 5-fu/lv for the treatment for patients with metastatic carcinoma of the colon or rectum whose disease has progressed during or within 6 months of completion of first line therapy with the combination of bolus 5 -fu/lv and irinotecan
Arimidex	anastrozole	3/5/2002	9/5/2002	Yes	for the adjuvant treatment of postmenopausal women with hormone receptor positive early breast cancer
Gleevec	imatinib mesylate	6/28/2002	12/20/2002	Yes	for the treatment of newly diagnosed adult patients with philadelphia chromosome positive chronic myeloid leukemia (cml)
Fuzeon	enfuvirtide	9/16/2002	3/13/2003	No	in combination with other antiretroviral agents for the treatment of hiv-1 infection in treatment experienced patients with evidence of hiv-1 replication despite ongoing antiretroviral therapy
Fabrazyme	agalsidase beta	6/23/2000	4/24/2003	No	for the treatment of fabry disease

© 2021 Sachs RE et al. *JAMA Health Forum.*

Iressa	gefitinib	8/5/2002	5/5/2003	No	as a monotherapy for the treatment of patients with locally advanced or metastatic non-small cell lung cancer after failure of both platinum-based and docetaxel chemotherapy
Velcade	bortezomib	1/21/2003	5/13/2003	No	for the treatment of multiple myeloma patients who have received at least two prior therapies and have demonstrated disease progression on the last therapy
Gleevec	imatinib mesylate	6/28/2002	5/20/2003	Yes	for the treatment of pediatric patients with ph+ chronic phase cml whose disease has recurred after stem cell transplant or who are resistant to interferon alpha therapy
Erbitux	cetuximab	8/14/2003	2/12/2004	No	as a single agent for the treatment of egfr-expressing, metastatic colorectal carcinoma in patients who are intolerant to irinotecan-based chemotherapy
Erbitux	cetuximab	8/14/2003	2/12/2004	No	in combination with irinotecan for the treatment of egfr-expressing metastatic colorectal carcinoma in patients who are refractory to irinotecan-based chemotherapy
Truvada	tenofovir disoproxil fumarate; emtricitabine	3/12/2004	8/2/2004	No	in combination with other antiretroviral agents for the treatment of hiv infection in adults
Alimta	pemetrexed disodium	11/4/2003	8/19/2004	No	as a single agent for the treatment of patients with locally advanced or metastataic non-small cell lung cancer after prior chemotherpy
Femara	letrozole	4/29/2004	10/29/2004	Yes	for the extended adjuvant treatment of early breast cancer in postmenopausal women who have received five years of adjuvant tamoxifen therapy
Tysabri	natalizumab	5/24/2004	11/23/2004	No	for the treatment of patients with relapsing forms of multiple sclerosis (ms) to reduce the frequency of clinical exacerbations
Levaquin	levofloxacin	11/12/2004	11/24/2004	Yes	for the treatment of inhalational anthrax (post-exposure)

© 2021 Sachs RE et al. *JAMA Health Forum.*

Clolar	clofarbine	3/30/2004	12/28/2004	No	for the treatment of pediatric patients 1 to 21 years old with relapsed or refractory acute lymphocytic leukemia after at least two prior regimens
Aptivus	tipranavir	12/22/2004	6/22/2005	No	co-administered with 200 mg of ritonavir for combination antiretroviral treatment of hiv-1 infected adult patients with evidence of viral replication, who are highly treatment-experienced or have hiv-1 strains resistant to multiple protease inhibitors
Arranon	nelarabine	4/29/2005	10/28/2005	No	for the treatment of patients with t-cell acute lymphoblastic leukemia and t-cell lymphoblastic lymphoma whose disease has not responded to or has relapsed following treatment with at least two chemotherapy regimens
Exjade	deferasirox	5/2/2005	11/2/2005	No	for the treatment of chronic iron overload due to blood transfusions in patients 2 years of age and older
Femara	letrozole	6/28/2005	12/28/2005	Yes	for the adjuvant treatment of postmenopausal women with hormone receptor positive early breast cancer
Sutent	sunitinib malate	8/11/2005	1/26/2006	No	for the treatment of advanced renal cell carcinoma
Thalomid	thalidomide	12/23/2003	5/25/2006	No	for the treatment of patients with newly diagnosed multiple myeloma
Prezista	darunavir	12/23/2005	6/23/2006	No	for the treatment of human immunodeficiency virus (hiv) infection in antiretroviral treatment-experienced adult patients, such as those with hiv-1 strains resistant to more than one protease inhibitor
Sprycel	dasatinib	12/28/2005	6/28/2006	No	for the treatment of chronic myeloid leukemia with resistance or intolerance to prior therapy including imatinib
Vectibix	panitumumab	3/29/2006	9/27/2006	No	for the treatment of egfr-expressing, metastatic colorectal carcinoma with disease progression on or following fluoropyrimidine-, oxaliplatin-, and

					irinotecan-containing chemotherapy regimens
Selzentry	maraviroc	12/20/2006	8/6/2007	No	for the treatment of patients with ccr5-tropic hiv-1
Isentress	raltegravir	4/13/2007	10/12/2007	No	in combination with other antiretroviral agents, for the treatment of hiv 1 infection in treatment-experienced adult patients who have evidence of viral replication and hiv-1 strains resistant to multiple antiretorviral agents
Tasigna	nilotinib	9/29/2006	10/29/2007	No	for the treatment of chronic phase (cp) and accelerated phase (ac) philadelphia chromosone positive chronic myelogenous leukemia(cml)in adult patients resistant to or intolerant to prior therapy that included gleevec (imatinib)
Intelence	etravirine	7/18/2007	1/28/2008	No	in combination with other antiretroviral agents for the treatment of hiv-1 iinfection in treatment-experienced adult patients who have evidence of viral replications and hiv-1 strains resistant to non-nucleloside reverse transcriptase inhibitor (nnrti) and other antiretroviral agent
Avastin	bevacizumab	5/24/2008	2/22/2008	Yes	new indication for use in combination with paclitaxel for the treatment of patients who have not received chemotherapy for metastatic her2 negative breast cancer
Levaquin	levofloxacin	7/5/2007	5/5/2008	Yes	to reduce the incidence or progression of disease following exposure to aerosolized bacillus anthracis in pediatric patients (>6 month of age and older)
Alimta	pemetrexed disodium	8/28/2007	9/26/2008	Yes	for the treatment of • nonsquamous non-small cell lung cancer: initial treatment incombination with cisplatin. (1.1)• nonsquamous non-small cell lung cancer as a single-agent after priorchemotherapy (1.2)

© 2021 Sachs RE et al. *JAMA Health Forum.*

Promacta	eltrombopag	12/19/2007	11/20/2008	No	for the treatment thrombocytopneia in patients with chronic immune (idiopathic) thrombocytopenia (itp) who have had an insufficient response to corticosteroids, immunoglobulins, or splenectomy
Gleevec	imatinib mesylate	6/24/2008	12/29/2008	Yes	for the adjuvant treatment of adult patients following complete gross resection of kit (cd117) positive gastrointestinal stromal tumors (gist)
Avastin	bevacizumab	11/3/2008	5/5/2009	Yes	for the treatment of glioblastoma with progressive disease following prior therapy
Folotyn	pralatrexate	3/24/2009	9/24/2009	No	for the treatment of relapsed or refractory periperal t-cell lymphoma
Arzerra	ofatumumab	1/30/2009	10/26/2009	No	for the treatment of patients with chronic lymphocytic leukemia (cll) refractory to fludarabine and alemtuzumab
Tykerb	lapatinib	3/31/2009	1/29/2010	Yes	tykerb in combination with letrozole is for the treatment of post menopausal women with hormone receptor positive metastatic breast cancer that overexpress the her2 receptor for whom hormonal therapy is indicated
Tasigna	nilotinib	12/21/2009	6/17/2010	Yes	for the treatment of newly diagnosed adult patients with philadelphia chromosome positive chronic myeloid leukemia in chronic phase
Sprycel	dasatinib	4/28/2010	10/28/2010	Yes	for the treatment of newly diagnosed adults with philadelphia chromosome-positive (ph+) cml in chronic phase
Afinitor	everolimus	4/30/2010	10/29/2010	Yes	for the treatment of subependymal giant cell astrocytoma (sega) associated with tuberous sclerosis (ts) who require therapeutic intervention but are not candidates for curative surgical resection
Makena	Hydroxyprogesterone caproate injection	4/20/2006	2/3/2011	No	to reduce the risk of preterm birth in women with a singleton pregnancy who have a history of singleton spontaneous preterm birth

© 2021 Sachs RE et al. *JAMA Health Forum.*

Istodax	romidepsin	12/17/2010	6/16/2011	Yes	the treatment of peripheral t-cell lymphoma (ptcl) in patients who have received at least one prior therapy
Adcetris	brentuximab vedotin	2/28/2011	8/19/2011	No	for the treatment of patients with hodgkin lymphoma after failure of autologous stem cell transplant (asct) or after failure of at least two prior multi-agent chemotherapy regimens in patients who are not asct candidates
Adcetris	brentuximab vedotin	2/28/2011	8/19/2011	Yes	for the treatment of patients with systemic anaplastic large cell lymphoma (salcl) after failure of at least one prior mulit-agent chemotherpay regimen
Xalkori	crizotinib	3/30/2011	8/26/2011	No	for the treatment of patients with locally advanced or metastatic non-small cell lung cancer (nsclc) that is anaplastic lymphoma kinase (alk)-positive as detected by an fda approved test
Ferriprox	deferiprone	1/30/2009	10/14/2011	No	for the treatment of transfusional iron overload due to thalassemia syndromes when current chelation therapy is inadequate
Afinitor	everolimus	12/19/2011	4/26/2012	Yes	treatment of adults with renal angiomyolipoma and tuberous sclerosis complex (tsc) not requiring immediate surgery
Kyprolis	carfilzomib	9/27/2011	7/20/2012	No	for the treatment of patients with multiple myeloma who have received at least two prior<br>therapies including bortezomib and an immunomodulatory agent and have demonstrated disease progression on or within 60 days of completion of the last therapy

© 2021 Sachs RE et al. *JAMA Health Forum.*

Marqibo	vincristine sulfate (liposomal)	7/12/2011	8/9/2012	No	for the treatment of adults with philadelphia (ph) chromosome negative (-) acute lymphoblastic leukemia (all) in second relapse or greater relapse or whose disease has progressed following two or greather treatment lines of anti-leukemia therapies
Afinitor Disperz	everolimus	2/29/2012	8/29/2012	No	for the treatment of pediatric and adult patients with tuberous sclerosis complex (tsc) for the treatment of subependymal giant cell astrocytoma (sega) that requires therapeutic intervention but cannot be curatively resected
Synribo	omacetaxine mepesuccinate	3/30/2012	10/26/2012	No	for the treatment of adult patients with chronic or accelerated phase chronic myeloid leukemia (cml) with resistance and/orintolerance to two or more tyrosine kinase inhibitors (tki)
Iclusig	ponatinib	9/27/2012	12/14/2012	No	for the treatment of adult patients with chronic phase, accelerated phase, or blast phase chronic myeloid leukemia (cml) that is resistant or intolerant to prior tyrosine kinase inhibitor therapy or philadelphia chromosome positive acute lymphoblastic leukemia (ph+all) that is resistant or intolerant to prior tyrosine kinase inhibitor therapy
Sirturo	bedaquiline	6/29/2012	12/28/2012	No	for the treatment of, as combination therapy, adults (≥ 18 years) with pulmonary multi-drug resistant tuberculosis (mdr-tb)

© 2021 Sachs RE et al. *JAMA Health Forum.*

Exjade	deferasirox	12/23/2011	1/23/2013	Yes	treatment of chronic iron overload in patients 10 years of age and older with non-transfusion dependent thalassemia syndromes and with a liver iron concentration of at least 5 mg fe/g dw and a serum ferritin greater than 300 mcg/l
Pomalyst	pomalidomide	4/10/2012	2/8/2013	No	for the treatment of patients with multiple myeloma who have received at least two prior therapies including lenalidomide and bortezomib and have demonstrated disease progression on or within 60 days of completion of the last therapy
Perjeta	pertuzumab	5/1/2013	9/30/2013	Yes	in combination with trastuzumab and docetaxel for the neoadjuvant treatment of patients with her2-positive, locally advanced, inflammatory, or early stage breast cancer (either greater than 2 cm in diameter or node positive) as part of a complete treatment regimen for early breast cancer
Imbruvica	ibrutinib	6/28/2013	11/13/2013	No	for the treatment of patients with mantle cell lymphoma (mcl)
Mekinist	trametinib	7/8/2013	1/8/2014	Yes	in combination with dabrafenib, is indicated for the treatment of patients with unresectable or metastatic melanoma with braf v600e or v600k mutations, as detected by an fda-approved test
Tafinlar	dabrafenib	7/9/2013	1/9/2014	Yes	in combination with trametinib, for the treatment of patients with unresectable or metastatic melanoma with braf v600e or v600k mutations, as detected by an fda-approved test

© 2021 Sachs RE et al. *JAMA Health Forum.*

Imbruvica	ibrutinib	6/28/2013	2/12/2014	No	for the treatment of patients with chronic lymphocytic leukemia (cll) who have received at least one prior therapy
Northera	droxidopa	9/28/2011	2/18/2014	No	for the treatment of orthostatic dizziness, lightheadedness, or the “feeling that you are about to black out” in adult patients with symptomatic neurogenic orthostatic hypotension caused by primary autonomic failure (parkinson's disease, multiple system atrophy, and pure autonomic failure), dopamine beta-hydroxylase deficiency, and non-diabetic autonomic neuropathy
Zykadia	ceritinib	12/24/2013	4/29/2014	No	for the treatmentof patients with anaplastic lymphoma kinase (alk)-positive metastatic non-small cell lung cancer (nsclc) who have progressed on or are intolerant to crizotinib
Beleodaq	belinostat	12/9/2013	7/3/2014	No	for the treatment of patients with relapsed or refractory peripheral t-cell lymphoma
Zydelig	idelalisib	9/11/2013	7/23/2014	No	for the treatment of relapsed follicular bcell non-hodgkin lymphoma (fl) in patients who have received at least two prior systemic therapies and relapsed small lymphocytic lymphoma (sll) in patients who have received at least two prior systemic therapies
Keytruda	pembrolizumab	11/22/2013	9/4/2014	No	for the treatment of patients with unresectable or metastatic melanoma and disease progression following ipilimumab and, if braf v600 mutation positive, a braf inhibitor
Blincyto	blinatumomab	9/19/2014	12/3/2014	No	for the treatment of philadelphia chromosome negative relapsed or refractory b-cell precursor acute lymphoblastic leukemia (all)

© 2021 Sachs RE et al. *JAMA Health Forum.*

Lynparza	olaparib	2/3/2014	12/19/2014	No	for patients with deleterious or suspected deleterious germline brca mutated advanced ovarian cancer who have been treated with three or more prior lines of chemotherapy
Opdivo	nivolumab	7/30/2014	12/22/2014	No	for the treatment of unresectable or metastatic melanoma and disease progression following ipilimumab and, if braf v600 mutation positive, a braf inhibitor
Ibrance	palbociclib	8/13/2014	2/3/2015	No	in combination with letrozole for the treatment of postmenopausal women with estrogen receptor(er)-positive, human epidermal growth factor receptor 2 (her2)-negative advanced breast cancer as initial endocrine-based therapy for their metastatic disease
Farydak	panobinostat	3/24/2014	2/23/2015	No	in combination with bortezomib (btz) and dexamethasone(dex) for the treatment of patients with multiple myeloma (mm) who have received at least 2 prior regimens, including bortezomib and an immunomodulatory agent
Jadenu	deferasirox	5/30/2014	3/30/2015	No	for the treatment of chronic iron overload in patients 10 years of age and older with nontransfusion-dependent thalassemia (ntdt) syndromes and with a liver iron concentration (lic) of at least 5 milligrams of iron per gram of liver dry weight (mg fe/g dw) and a serum ferritin greater than 300 mcg/l
Jadenu	deferasirox	5/30/2014	3/30/2015	No	for the treatment of chronic iron overload due to blood transfusions (transfusional hemosiderosis) in patients 2 years of age and older

© 2021 Sachs RE et al. *JAMA Health Forum.*

Opdivo	nivolumab	3/30/2015	9/30/2015	Yes	in combination with ipilimumab, for the treatment of patients with braf v600 wildtype, unresectable or metastatic melanoma
Keytruda	pembrolizumab	4/2/2015	10/2/2015	Yes	for the treatment of patients with metastatic, pd-l1 positive, non-small cell lung cancer (nsclc), as determined by an fdaapproved test, with disease progression on or after platinum-containing chemotherapy
Praxbind	idarucizumab	2/19/2015	10/16/2015	No	for the treatment of patients treated with pradaxa® when reversal of the anticoagulant effects of dabigatran is needed for emergency surgery/urgent procedures and in life-threatening or uncontrolled bleeding
Tagrisso	osimertinib	6/5/2015	11/13/2015	No	for the treatment of patients with metastatic epidermal growth factor receptor (egfr) t790m mutation-positive-non-small-cell lung cancer (nsclc), as detected by an fdaapproved test, who have progressed on or after egfr tki therapy
Darzalex	daratumumab	7/9/2015	11/16/2015	No	myeloma who have received at least 3 prior lines of therapy including a proteasome inhibitor and an immunomodulatory agent or are double refractory to a proteasome inhibitor and an immunomodulatory agent
Alecensa	alectinib	7/6/2015	12/11/2015	No	for the treatment of patients with anaplastic lymphoma kinase (alk)-positive metastatic nonsmall cell lung cancer (nsclc), who have progressed on or are intolerant to crizotinib

© 2021 Sachs RE et al. *JAMA Health Forum.*

Opdivo	nivolumab	7/23/2015	1/23/2016	Yes	1) in combination with ipilimumab for the treatment of pateints with unresectable or metastatic melanoma to remove the restriction for the treatment of only patients wiht braf wild-type melanoma, 2) as a single agent for the treatment of patients with braf v600 mutation positive, unresectable or metastatic melanoma to remove the restriction that such patients should have disease progression following ipilimumab and a braf inhibitor
Provayblue	methylene blue	10/9/2015	4/8/2016	No	for the treatment of pediatric and adult patients with acquired methemoglobinemia
Venclexta	venetoclax	10/29/2015	4/11/2016	No	for the treatment of patients with chronic lymphocytic leukemia (cll) with 17p deletion, as detected by an fda approved test, who have received at least one prior therapy
Opdivo	nivolumab	3/1/2016	5/17/2016	Yes	for the treatment of classical hodgkin lymphoma that has relapsed or progressed after autologous hematopoietic stem cell transplantation (hsct) and posttransplantation brentuximab vedotin.
Tecentriq	atezolizumab	1/12/2016	5/18/2016	No	for the treatment of patients with locally advanced or metastatic urothelial carcinoma who have disease progression during or following platinum-containing chemotherapy or have disease progression within 12 months of neoadjuvant or adjuvant treatment with platinum-containing chemotherapy

© 2021 Sachs RE et al. *JAMA Health Forum.*

Ocaliva	obeticholic acid	6/29/2015	5/27/2016	No	for the treatment of primary biliary cholangitis (pbc) in combination withursodeoxycholic acid (udca) in adults with an inadequate response to udca, or as monotherapy in adults unable to tolerate udca
Keytruda	pembrolizumab	2/9/2016	8/5/2016	Yes	for the treatment of patients with recurrent or metastatic squamous cell carcinoma of the head and neck with disease progression on or after platinum-containing chemotherapy
Exondys 51	eteplirsen	6/26/2015	9/19/2016	No	for the treatment of duchenne muscular dystrophy (dmd) in patients who have a confirmed mutation of the dmd gene that is amenable to exon 51 skipping
Lartruvo	olaratumab	2/24/2016	10/19/2016	No	in combination with doxorubicin for the treatment of adult patients with soft tissue sarcoma (sts) with a histologic subtype for which an anthracycline-containing regimen is appropriate and which is not amenable to curative treatment with radiotherapy or surgery
Rubraca	rucaparib	6/23/2016	12/19/2016	No	for the treatment of patients with deleterious brca mutation (germline and/or somatic) associated advanced ovarian cancer who have been treated with two or more chemotherapies
Imbruvica	ibrutinib	9/23/2016	1/18/2017	Yes	for the treatment of patients with marginal zone lymphoma (mzl) who require systemic therapy and have received at least one prior anti-cd20-based therapy

© 2021 Sachs RE et al. *JAMA Health Forum.*

Opdivo	nivolumab	9/2/2016	2/2/2017	Yes	for the treatment of locally advanced or metastatic urothelial carcinoma who: • have disease progression during or following platinum-containing chemotherapy • have disease progression within 12 months of neoadjuvant or adjuvant treatment with platinum-containing chemotherapy.
Keytruda	pembrolizumab	9/15/2016	3/14/2017	Yes	for the treatment of adult and pediatric patients with refractory classical hodgkin lymphoma, or who have relapsed after 3 or more prior lines of therapy
Bavencio	avelumab	9/23/2016	3/23/2017	No	for the treatment of adults and pediatric patients 12 years and older with metastatic merkel cell carcinoma
Tecentriq	atezolizumab	10/31/2016	4/17/2017	Yes	patients with locally advanced or metastatic urothelial carcinoma who are not eligible for cisplatin-containing chemotherapy, or have disease progression during or following any platinum-containing chemotherapy, or within 12 months of neoadjuvant or adjuvant chemotherapy
Opdivo	nivolumab	12/9/2016	4/25/2017	Yes	for the treatment of adult patients with classical hodgkin lymphoma that has relapsed or progressed after: • autologous hematopoietic stem cell transplantation (hsct) and brentuximab vedotin, or • 3 or more lines of systemic therapy that includes autologous hsct.

© 2021 Sachs RE et al. *JAMA Health Forum.*

Alunbrig	brigatinib	8/29/2016	4/28/2017	No	for treatment of patients with anaplastic lymphoma kinase (alk)-positive metastatic nonsmall cell lung cancer (nsclc) who have progressed on or are intolerant to crizotinib
Imfinzi	durvalumab	10/13/2016	5/1/2017	No	for the treatment of patients with locally advanced or metastatic urothelial carcinoma who have disease progression during or following platinum-containing chemotherapy or have disease progression within 12 months of neoadjuvant or adjuvant treatment with platinum-containing chemotherapy
Bavencio	avelumab	12/27/2016	5/9/2017	No	for the treatment of patients with locally advanced or metastatic urothelial carcinoma (uc) who: have disease progression during or following platinum-containing chemotherapy or who have disease progression within 12 months of neoadjuvant or adjuvant treatment with platinum-containing
Keytruda	pembrolizumab	11/10/2016	5/10/2017	Yes	in combination with pemetrexed and carboplatin, for the first-line treatment of patients with metastatic non-squamous, nonsmall cell lung cancer
Keytruda	pembrolizumab	12/14/2016	5/18/2017	Yes	for the treatment of patients with locally advanced or metastatic urothelial carcinoma who are not eligible for cisplatin-containing chemotherapy

© 2021 Sachs RE et al. *JAMA Health Forum.*

Jadenu sprinkle	deferasirox	7/21/2016	5/18/2017	No	for the treatment of chronic iron overload in patients 10 years of age and older with nontransfusion- dependent thalassemia (ntdt) syndromes and with a liver iron concentration (lic) of at least 5 milligrams of iron per gram of liver dry weight (mg fe/g dw) and a serum ferritin greater than 300 mcg/l
Jadenu sprinkle	deferasirox	7/22/2016	5/19/2017	No	for the treatment of chronic iron overload due to blood transfusions (transfusional hemosiderosis) in patients 2 years of age and older
Keytruda	pembrolizumab	9/8/2016	5/23/2017	Yes	for the treatment of adult and pediatric patients with unresectable or metastatic, microsatellite instability-high (msi-h) or mismatch repair deficient solid tumors that have progressed following prior treatment and who have no satisfactory alternative treatment options, or metastatic microsatellite instability-high (msh-h) or mismatch repair deficient colorectal cancer that has progressed following treatment with a fluoropyrimidine, oxaliplatin, and irinotecan
Opdivo	nivolumab	2/2/2017	7/31/2017	Yes	for the treatment of adult and pediatric patients 12 years and older with microsatellite instability-high (msi-h) or mismatch repair deficient (dmmr) metastatic colorectal cancer (crc) that has progressed following treatment with a fluoropyrimidine, oxaliplatin, and irinotecan

© 2021 Sachs RE et al. *JAMA Health Forum.*

Benznidazole	benznidazole	12/29/2016	8/29/2017	No	for the treatment of chagas disease (american trypanosomiasis), caused by trypanosoma cruzi, in pediatric patients 2 to 12 years of age
Aliqopa	copanlisib	3/16/2017	9/14/2017	No	for the treatment of adult patients with relapsed follicular lymphoma (fl) who have received at least two prior systemic therapies
Keytruda	pembrolizumab	3/22/2017	9/22/2017	Yes	for the treatment of patients with recurrent locally advanced or metastatic gastric or gastroesophageal junction adenocarcinoma whose tumors express pd-l1 [combined positive score (cps) ≥1] as determined by an fdaapproved test, with disease progression on or after two or more prior lines of therapy including fluoropyrimidine- and platinumcontaining chemotherapy and if appropriate, her2/neu targeted therapy
Opdivo	nivolumab	3/24/2017	9/22/2017	Yes	for the treatment of patients with hepatocellular carcinoma who have been previously treated with sorafenib
Calquence	acalabrutinib	6/13/2017	10/31/2017	No	for the treatment of adult patients with mantle cell lymphoma (mcl) who have received at least one prior therapy
Bosulif	bosutinib	6/26/2017	12/19/2017	Yes	for the treatment of adult patients with newly diagnosed chronic phase (cp) philadelphia chromosome positive chronic myelogenous leukemia (ph+ cml)
Imbruvica	ibrutinib	8/31/2017	2/16/2018	No	for the treatment of adult patients with mantle cell lymphoma (mcl) who have received at least one prior therapy

© 2021 Sachs RE et al. *JAMA Health Forum.*

Blincyto	blinatumomab	9/29/2017	3/29/2018	Yes	for the treament of b-cell precursor acute lymphoblastic leukemia (all) in first or second completed remission with minimal residual disease (mrd) greater or equal to 0.1% in adults and children
Alimta	pemetrexed disodium	8/3/2017	6/4/2018	Yes	in combination with pembrolizumab and carboplatin, for the first-line treatment of metastatic, non-squamous, non-small cell lung cancer
Keytruda	pembrolizumab	12/28/2017	6/12/2018	Yes	for the treatment of patients with recurrent or metastatic cervical cancer with disease progression on or after chemotherpay whoses tumors express pd-l1 (cps > 1) as determined by an fda-approved test
Keytruda	pembrolizumab	10/3/2017	6/13/2018	Yes	for the treament of adult and pediatric patients with refractory primary mediastinal large b-cell lymphoma (pmbcl), or who have relapsed after 2 or more prior lines of therapy
Opdivo	nivolumab	1/10/2018	7/10/2018	Yes	in combination with ipilimumab for the treatment of adults and pediatric patients 12 years and older with microsatellite instability-high (msi h) or dna mismatch repair deficient (dmmr), metastatic colorectal cancer that has progressed following treatment with a fluoropyrimidine, oxaliplatin, and irinotecan

© 2021 Sachs RE et al. *JAMA Health Forum.*

Yervoy	ipilimumbad	1/10/2018	7/10/2018	Yes	in combination with nivolumab for the treatment of adults and pediatric patients 12 years and older with microsatellite instability-high (msi h) or dna mismatch repair deficient (dmmr), metastatic colorectal cancer that has progressed following treatment with a fluoropyrimidine, oxaliplatin, and irinotecan
Galafold	migalastat	12/13/2017	8/10/2018	No	for the treatment of adults with a confirmed diagnosis of fabry disease and an amenable galactosidase alpha gene (gla) variant based on in vitro assay data
Opdivo	nivolumab	2/16/2018	8/16/2018	Yes	for the treatment of patients with metastatic small cell lung cancer (sclc) with progression after platinum-based chemotherapy and at least one other line of therapy
Copiktra	duvelisib	2/5/2018	9/24/2018	No	for the treatment of adult patients with relapsed or refractory follicular lymphoma (fl) after at least two prior systemic therapies

© 2021 Sachs RE et al. *JAMA Health Forum.*

Arikayce	amikacin liposome inhalation suspension	3/28/2018	9/28/2018	No	arikayce is an aminoglycoside antibacterial indicated in adults who have limited or no alternative treatment options, for the treatment of mycobacterium avium complex (mac) lung disease as part of a combination antibacterial drug regimen in patients who do not achieve negative sputum cultures after a minimum of 6 consecutive months of a multidrug background regimen therapy. as only limited clinical safety and effectiveness data for arikayce are currently available, reserve arikayce for use in adults who have limited or no alternative treatment options. this drug is indicated for use in a limited and specific population of patients. this indication is approved under accelerated approval based on achieving sputum culture conversion (defined as 3 consecutive negative monthly sputum cultures) by month 6. clinical benefit has not yet been established
Lorbrena	lorlatinib	12/5/2017	11/2/2018	No	for the treatment of patients with anaplastic lymphoma kinase (alk)-positive metastatic nonsmall cell lung cancer (nsclc) whose disease has progressed on: • crizotinib and at least one other alk inhibitor for metastatic disease; or • alectinib as the first alk inhibitor therapy for metastatic disease; or • ceritinib as the first alk inhibitor therapy for metastatic disease

© 2021 Sachs RE et al. *JAMA Health Forum.*

Keytruda	pembrolizumab	5/9/2018	11/9/2018	Yes	for the treatment of patients with hepatocellular carcinoma who have been previously treated with sorafenib
Venclexta	venetoclax	6/25/2018	11/21/2018	Yes	venclexta in combination with azacitidine or decitabine or low-dose cytarabine for the treatment of newly-diagnosed acute myeloid leukemia (aml) in adults who are age 75 years or older, or who have comorbidities that preclude use of intensive induction chemotherapy
Vitrakvi	larotrectinib	3/26/2018	11/26/2018	No	for the treatment of adult and pediatric patients with solid tumors that have a neurotrophic receptor tyrosine kinase (ntrk) gene fusion without a known acquired resistance mutation; are metastatic or where surgical resection is likely to result in severe morbidity; and have no satisfactory alternative treatments or that have progressed following treatment
Keytruda	pembrolizumab	6/29/2018	12/19/2018	Yes	for the treatment of adult and pediatric patients with recurrent locally advanced or metastatic merkel cell carcinoma
Tecentriq	atezolizumab	9/12/2018	3/8/2019	Yes	in combination with paclitaxel protein-bound for the treatment of adult patients with unresectable locally advanced or metastatic triple-negative breast cancer (tnbc) whose tumors express pd-l1 (pd-l1 stained tumorinfiltrating immune cells [ic] of any intensity covering ≥ 1% of the tumor area), as determined by an fda-approved test

© 2021 Sachs RE et al. *JAMA Health Forum.*

Balversa	erdafitinib	9/18/2018	4/12/2019	No	for the treatment of adult patients with locally advanced or metastatic urothelial carcinoma (muc), that has: susceptible fgfr3 or fgfr2 genetic alterations and progressed during or following at least one line of prior platinum containing chemotherapy, including within 12 months of neoadjuvant or adjuvant platinum containing chemotherapy
Polivy	polatuzumab vedotin-piiq	12/19/2018	6/10/2019	No	in combination with bendamustine and rituximab product, for the treatment of adult patients with relapsed or refractory diffuse large b-cell lymphoma, not otherwise specified, after at least two prior therapies
Keytruda	pembrolizumab	12/17/2018	6/17/2019	Yes	for the treatment of patients with metastatic small cell lung cancer with disease progression on or after platinum-based chemotherapy and at least one other prior line of therapy
Xpovio	selinexor	8/6/2018	7/3/2019	No	in combination with dexamethasone for the treatment of adult patients with relapsed or refractory multiple myeloma (rrmm) who have received at least four prior therapies and whose disease is refractory to at least two proteasome inhibitors, at least two immunomodulatory agents, and an anti-cd38 monoclonal antibody

© 2021 Sachs RE et al. *JAMA Health Forum.*

Rozlytrek	entrectinib	12/18/2018	8/15/2019	No	for the treatment of adult and pediatric patients 12 years of age and older with solid tumors that have a neurotrophic tyrosine receptor kinase (ntrk) gene fusion without a known acquired resistance mutation, are metastatic or where surgical resection is likely to result in sever morbidity, and have progressed following treatment or have satisfactory alternative therapy
Keytruda	pembrolizumab	6/17/2019	9/17/2019	Yes	in combination with lenvatinib, indicated for the treatment of patients with advanced endometrial carcinoma that is not microsatellite instability-high (msi-h) or mismatch repair deficient (dmmr), who have disease progression following prior systemic therapy and are not candidates for curative surgery or radiation
Lenvima	lenvatinib	6/17/2019	9/17/2019	Yes	in combination with pembrolizumab, indicated for the treatment of patients with advanced endometrial carcinoma that is not microsatellite instability-high (msi-h) or mismatch repair deficient (dmmr), who have disease progression following prior systemic therapy and are not candidates for curative surgery or radiation
Oxbryta	voxelotor	6/26/2019	11/25/2019	No	for the treatment of sickle cell disease in adults and pediatric patients 12 years of age and older
Opdivo	nivolumab	9/10/2019	3/10/2020	Yes	in combination with ipilimumab, for the treatment of patients with hepatocellular carcinoma (hcc) who have been previously treated with sorafenib

© 2021 Sachs RE et al. *JAMA Health Forum.*

Yervoy	ipilimumbad	9/10/2019	3/10/2020	Yes	in combination with nivolumab, for the treatment of patients with hepatocellular carcinoma (hcc) who have been previously treated with sorafenib
Keytruda	pembrolizumab	4/15/2020	4/28/2020	Yes	provides for an alternate dose/schedule of 400mg every 6 weeks for adult patients with recurrent locally advanced or metastatic esophageal cancer with disease progression on or after 2 or more prior lines of systemic therapy
Keytruda	pembrolizumab	4/14/2020	4/28/2020	Yes	provides for an alternate dose/schedule of 400mg every 6 weeks for adult patients with recurrent or metastatic head and neck squamous cell carcinoma (hnscc) with disease progression on or after platinum-containing chemotherapy
Keytruda	pembrolizumab	4/14/2020	4/28/2020	Yes	provides for an alternate dose/schedule of 400mg every 6 weeks for adult patients with bcg-unresponsive, high risk, non-muscle invasive bladder cancer (nmibc) with carcinoma in-situ (cis) with or without papillary tumors who are ineligible for or have elected not to undergo cystectomy
Keytruda	pembrolizumab	4/14/2020	4/28/2020	Yes	provides for an alternate dose/schedule of 400mg every 6 weeks for adult patients with renal cell cancer (rcc)
Pomalyst	pomalidomide	11/14/2019	5/14/2020	Yes	for the treatment of adult patients with aidsrelated kaposi sarcoma (ks) after failure of highly active antiretroviral therapy (haart)
Pomalyst	pomalidomide	11/19/2019	5/14/2020	Yes	for the treatment of kaposi’s sarcoma in patients who are hiv-negative

© 2021 Sachs RE et al. *JAMA Health Forum.*

Rubraca	rucaparib	11/15/2019	5/15/2020	Yes	for the treatment of adult patients with a deleterious brca mutation (germline and/or somatic)-associated metastatic castrationresistant prostate cancer who have been treated with androgen receptor-directed therapy and a taxane-based chemotherapy
Keytruda	pembrolizumab	12/16/2019	6/16/2020	Yes	for the treatment of adult and pediatric patients with unresectable or metastatic tumor mutational burden-high (tmb-h) [≥10 mutations/megabase (mut/mb)] solid tumors, as determined by an fda-approved test, that have progressed following prior treatment and who have no satisfactory alternative treatment options
Xpovio	selinexor	12/23/2019	6/22/2020	Yes	for the treatment of adult patients with relapsed or refractory diffuse large b-cell lymphoma (dlbcl), not otherwise specified, including dlbcl arising from follicular lymphoma, after at least 2 lines of systemic therapy
Keytruda	pembrolizumab	6/12/2020	6/24/2020	Yes	provides for an alternate dosage regimen of 400 mg every 6 weeks for adult patients with recurrent or metastatic cutaneous squamous cell carcinoma (cscc) that is not curable by surgery or radiation
Keytruda	pembrolizumab	5/28/2020	11/13/2020	Yes	in combination with chemotherapy, for the treatment of patients with locally recurrent unresectable or metastatic triple negative breast cancer whose tumors express pd-l1 [combined positive score (cps) ≥10] as determined by an fda approved test

© 2021 Sachs RE et al. *JAMA Health Forum.*

eTable 3. Sensitivity Analysis of Spending and Utilization of Drugs With Accelerated Approval Excluding Drugs With Non–Accelerated Approval and Accelerated Approval Indications

Year	Accelerated Approval Drugs			All Drugs			% Spending on AA Drugs	% AA Prescriptions
	Gross Spending	Net Spending	Prescriptions	Gross Spending	Net Spending	Prescriptions		
2015	3,545,560,064	1,805,901,440	2,193,438	62,192,988,160	34,268,338,176	695,878,528	5.27	0.32
2016	3,963,640,320	2,027,342,848	2,013,644	68,715,900,928	33,533,360,128	743,912,512	6.05	0.27
2017	4,083,736,832	2,099,847,424	1,690,965	70,873,137,152	32,247,279,616	764,205,056	6.51	0.22
2018	3,914,957,568	2,015,504,000	1,224,472	67,486,158,848	27,331,895,296	740,084,544	7.37	0.17
2019	3,908,120,832	2,101,007,744	979,818	67,743,686,656	30,010,454,016	698,923,136	7.00	0.14

Note: Products that have non-AA and AA indications in separate or the same approval year have been excluded, including Sutent, Zydelig, Copiktra, Rozlytrek, Thalomid, Alimta, Ethyol, Arimidex, Femara, Avastin, Tykerb, Afinitor, Istodax, Perjeta, Tafinlar, Meknist, Bosulif, Yervoy, and Lenvima.

eTable 4. “Non-Medicaid” Amount Reimbursed as a Percentage of Total Amount Reimbursed

Year	AA Drugs	All Outpatient Prescription Drugs
2015	4.71%	4.18%
2016	6.34%	4.35%
2017	8.89%	6.10%
2018	9.67%	6.69%
2019	7.19%	4.48%

© 2021 Sachs RE et al. *JAMA Health Forum.*
